# Dr. Dennis D. Loop

**Published:** 1904-05

**Authors:** 


Dr. DENNIS D. LOOP, of North East, Pa., died at his home, April 2, 1904, of apoplexy, aged 76 years. He graduated at the University of Buffalo in 1864, and served for many years as a curator of that institution.

